# An enzymatically controlled mucoadhesive system for enhancing flavour during food oral processing

**DOI:** 10.1038/s41538-019-0043-y

**Published:** 2019-07-01

**Authors:** Vlad Dinu, Arthur Gadon, Katherine Hurst, Mui Lim, Charfedinne Ayed, Richard B. Gillis, Gary G. Adams, Stephen E. Harding, Ian D. Fisk

**Affiliations:** 1 National Centre for Macromolecular Hydrodynamics, School of Biosciences, University of Nottingham, Sutton Bonington Campus, Leicestershire, UK; 2 Division of Food, Nutrition and Dietetics, School of Biosciences, University of Nottingham, Sutton Bonington Campus, Leicestershire, UK; 3 School of Health Sciences, Faculty of Medicine and Health Sciences, Queen’s Medical Centre, Clifton Boulevard, Nottingham, UK; 4 Universitetet i Oslo, Postboks 6762, St. Olavs plass, 0130 Oslo, Norway

**Keywords:** Biopolymers, Biomaterials - proteins

## Abstract

While a good mucoadhesive biopolymer must adhere to a mucus membrane, it must also have a good unloading ability. Here, we demonstrate that the biopolymer pullulan is partially digested by human salivary α-amylase, thus acting as a controlled release system, in which the enzyme triggers an increased release of flavour. Our oral processing simulations have confirmed an increase in the bioavailability of aroma and salt compounds as a function of oral pullulan degradation, although the release kinetics suggest a rather slow process. One of the greatest challenges in flavour science is to retain and rapidly unload the bioactive aroma and taste compounds in the oral cavity before they are ingested. By developing a cationic pullulan analogue we have, in theory, addressed the “loss through ingestion” issue by facilitating the adhesion of the modified polymer to the oral mucus, to retain more of the flavour in the oral cavity. Dimethylaminoethyl pullulan (DMAE-pullulan) was synthesised for the first time, and shown to bind submaxillary mucin, while still retaining its susceptibility to α-amylase hydrolysis. Although DMAE-pullulan is not currently food grade, we suggest that the synthesis of a sustainable food grade alternative would be a next generation mucoadhesive targeted for the oral cavity.

## Introduction

Mucadhesion describes the ability of a biochemical material to adhere to a mucosal membrane^1,2^ and has been a subject of research for the food industry and academia in recent years.^3–5^ However, it still remains a loosely understood and poorly applied concept. Several theories were proposed to describe mucoadhesion, particularly the wetting theory, mechanical interlocking, electron transfer, adsorption and fracture theories.^6^ Its main clinical relevance is to enhance drug loading capacity and residence time at tissues of interest. However, a good mucoadhesive must also have a very good unloading capability at the site of action.^2^ Oral mucoadhesion has recently attracted the attention of the food industry with regard to flavour maximisation during oral processing, particularly in “healthy” low calorie reformulated foods. This is because sensory properties, such as texture and flavour are the two most important factors impacting consumer choice, after cost^7,8^ however, mucoadhesion targeted at the oral cavity does not come without complications. The fate of the flavour perceived during oral processing will be determined by the rate of release of aroma and taste compounds from the salivary bolus and availability at aroma and taste receptors. The vast majority of flavour, alongside other bioactive compounds, are rapidly lost through ingestion and are therefore not available for perception.

Food grade biopolymers have always been an attractive option for the food industry. Many of them are anionic polysaccharides which have widely been applied as stabilising agents and thickeners. While many research groups have tried to characterise and compare their mucoadhesive properties,^4,5^ there is limited evidence to suggest that anionic polysaccharides are, in chemical terms, mucoadhesive. In order to understand the fundamental molecular processes involved in adhesion, we need to better understand the physico-chemical composition of mucus, which consists of an anionic mucin glycoprotein as its main structural component. Within the saliva, mucin is identified as the second most abundant component, after salivary α-amylase which varies upon stimulation.^9^ It is characterised as having much lower molecular weights (<500 kilo Daltons, kDa) and lower degree of glycosylation (~60%) as compared to gastric, intestinal or colonic mucins. Oral and salivary mucins consist of gel forming mucins derived from the MUC5B gene and low molecular weight, soluble mucins encoded by MUC7 genes.^10^ However, it is very difficult to obtain human salivary mucins in any useful quantity for performing mucoadhesive experiments. Therefore, in our study, as in most oral formulation research, mucin from bovine salivary/submaxillary glands (BSM) is employed as a close surrogate for its human equivalent.

Like other mucins, submaxillary mucins have an amino acid domain rich in serine and threonine that forms a bridge with the hydroxyl groups of the N-acetylgalactosamine residues of the carbohydrate fraction.^11,12^ The carbohydrate region consists of up to five different monosaccharides, such as sialic acids, galactose, fucose, N-acetylglucosamine and N-acetylgalactosamine. They can form weak hydrophobic interactions at their carbonyl and methyl groups, or can form electrostatic interactions via carboxylic acids or through the sulphate groups of the protein region.^2,11,12^ As a result of the negatively charged overall configuration of mucins, neutral and anionic food biopolymers such as hydroxypropylmethyl cellulose (HPMC), carboxymethyl cellulose (CMC), pectin, alginate, guar, carrageenan or xanthan cannot chemically interact with mucus under the dilute solution conditions of the bolus. While these polysaccharides can mix in the aqueous environment, and physically interact to define the rheology and tribology of the bolus, they lack the molecular ability to bind to the mucus membranes. Yet, various food polysaccharides are still considered mucoadhesive because they have been shown to extend the residence time and release kinetics of bioactive compounds, as a result of the physical and chemical properties of the food thickener i.e. high viscosity, gelation. However, the process by which these anionic hydrocolloids increase flavour intensity is still a matter of debate, whether it is a chemical or a purely rheological mechanism.

By contrast, polycations such as chitosan (p*Ka* ~5.5–7), have extensively been studied for their ability to form strong mucoadhesive electrostatic interactions with mucins, however chitosan applications are limited.^13^ While chitosan was found useful in mucoadhesive applications targeted at the gastro-intestinal region, its applications in the oral cavity are restricted, as chitosan is so strongly charged that it can precipitate mucins and other functional glycoproteins present in the saliva.^3,13^ Protein precipitation is also attributed to an unpleasant and astringent mouthfeel response, thereby negatively modifying the organoleptic properties of food.^14^ Besides, chitosan mucoadhesives are limited in their ability to “unload”, since a large proportion of the bioactive molecule remains trapped in the mucus/chitosan complex and is passed along the alimentary canal. Thiomers or thiolated polymers are an example of a more recent development of mucoadhesive formulations. They are principally synthesised by coupling thiol containing functional groups (SH), capable of forming stable hydrogen bonds with sulphate rich protein domains in mucin.^15^ However, the use or sulphur containing polymers is limited in flavour applications.

For food applications, there is a need to develop a tasteless, non-toxic and milder mucoadhesive, which has a good loading capacity, but which must also be able to unload the flavour compounds during mastication or during ingestion (via retronasal olfaction). Diethylaminoethyl-dextran or DEAE-Dextran, is an example of a much milder mucoadhesive that was shown to interact with mucin.^16^ However, its mucoadhesive properties were too modest considering the high charge density of the modified polymer. It was suggested that the α(1–3) branches of dextran and the presence of ethyl groups limit the access of the charged amino groups for the sialic acid groups of mucin, due to steric hindrance.

The polysaccharide pullulan, is produced by bacterial fermentation using *Aureobasidium pullulans*^17^ and is particularly used in Asia, as a partial replacement for starch as a low calorie ingredient in food and drink.^18^ It forms clear, odourless and tasteless solutions which do not gel, but can form transparent and oxygen impermeable films upon drying. Due to its film forming properties, it has been extensively used as a coating agent in confectionery, edible films, as well as a replacement for gelatin in medicinal applications.^18^ The use of pullulan has strong potential for encapsulation and release of flavour compounds due to its quick dissolution properties. For example, used as a breath freshener due to its ability to dissolve rapidly on consumption, and release the bound menthol molecules.^18^

It is a linear polymer consisting of α(1–4) linked maltotriose and infrequent maltotetraose units, linked together by α(1–6) glycosidic bonds. Previous studies suggested that some α-amylases are able to digest the polysaccharide at its maltotetraose units, thus rendering the polymer partially hydrolysed.^19^ It has previously been established that at equivalent polymer viscosities, starch thickened products have a good flavour and taste profile, and this is partially due to the decrease in viscosity in the mouth, resulting from salivary α-amylase digestion. In a comparable way, it is suggested that the partial in-vivo degradation of pullulan would result in an increased release of flavour, similar to starch based ingredients, which has been shown previously to enhance perception as a result of an increase in the concentration of volatile aroma compounds reaching the olfactory receptors.^20^ Thus, the polymer is expected to act as a controlled release excipient of aroma and taste molecules, in which salivary α-amylase releases actives close to the point of perception. Our hypothesis is that the synthesis of a mild pullulan mucoadhesive would reduce the loss of flavour through ingestion by increasing adhesion to the oral surface along with associated flavour compounds, provided the cationic polymer does not interfere with the normal functioning of the enzyme.

In the present study, we tested whether pullulan can be hydrolysed by human salivary α-amylase. Then, we evaluated its ability to modify flavour and salt release from model and real food systems by using Gas Chromatography-Mass Spectrometry (GC–MS), Atmospheric Pressure Chemical Ionization- Mass Spectrometry (APCI-MS), and also conductivity analysis using the INSENT^TM^ electronic tongue tasting system (E-tongue) and a standard conductivity probe. Then we synthesised a cationic pullulan analogue, dimethylaminoethyl pullulan (DMAE-pullulan), which was confirmed by Fourier-transform infrared spectroscopy (FT-IR). The newly synthesised polymer was subsequently evaluated for its mucoadhesive ability using a range of matrix/column free hydrodynamic techniques such as: Viscometry, Dynamic Light Scattering (DLS) and Sedimentation Velocity- Analytical ultracentrifugation (SV-AUC). To the best of the authors’ knowledge, this is the first synthesis of DMAE-pullulan. The advantage of dimethylaminoethyl compared to previously characterised diethylaminoethyl (DEAE) synthesis, is that the shorter methyl groups, as opposed to the ethyl groups, may increase the availability of the positively charged amino groups to the negatively charged carbohydrate residues of mucin.

## Results

### The impact of pullulan hydrolysis by α-amylase on flavour release

**Fig. 1:**
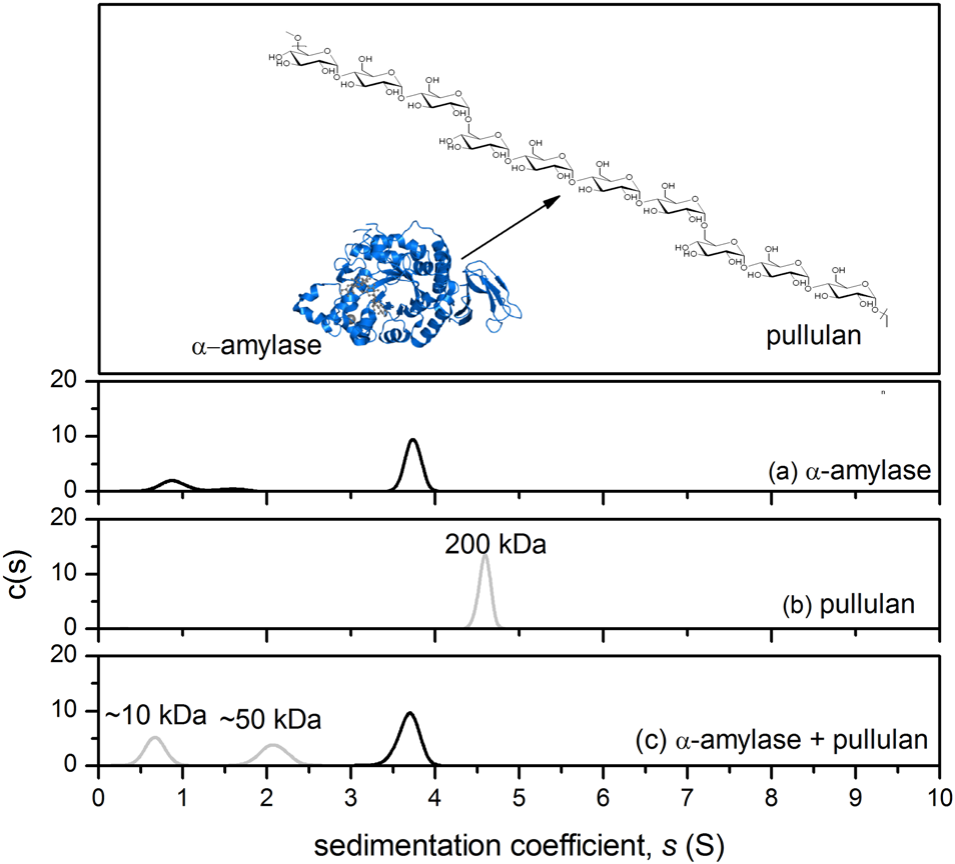
Structural representation of pullulan showing the its tetrasaccharide units which can be hydrolysed by α-amylase (top); and the sedimentation velocity- c(s) analysis (bottom), showing the sedimentation coefficient distributions of α-amylase **a**, 200 kDa pullulan standard **b**, and the result of their interaction **c**. A constant concentration of 1 mgmL^−1^ was used for the α-amylase and pullulan controls, and the mixture. Note that some of the material at ~0.8 S is also present in the α-amylase control, therefore some of it will contribute to an overestimate of peak ~0.8 S in the mixture. Rotor speed: 45,000 rpm (130,000 × *g*), 20.0 °C

Pullulan consists primarily of α(1–4) linked trisaccharide units linked together by α(1–6) glyosidic bonds (Fig. 1). However, depending on the fermentation conditions, the linear polymer has been found to contain up to 6% tetrasaccharide units, allowing access to the active site of α-amylase to hydrolyse the polymer^19^ (Fig. 1-top). We employed an SV-AUC experiment to analyse the resulting interaction between human salivary α-amylase (HSA) and pullulan. The analysis was performed using highly purified 200 kDa molecular weight pullulan standard, which yielded a single, monodisperse peak at ~4.6S (Fig. 1b). The addition of α-amylase revealed the formation of two distinct degradation fragments corresponding to a major peak at ~2S and a minor peak at ~0.8S (Fig. 1c). Note that a proportion of the monodisperse peak at ~0.8S is partly due to the presence of the smaller component present in the α-amylase control (Fig. 1a). The relative molecular weight for the digested fractions are approximated using 10 and 49 kDa molecular weight pullulan standards. For the rest of the investigation, we used an unfractionated, food grade 200 kDa commercially available source of pullulan. Next, an experiment was employed to determine the ability of HSA to digest the commercial product. For this we used dynamic light scattering (DLS) to examine changes in the molecular hydrodynamic size of pullulan before and after the addition of α-amylase.

**Fig. 2:**
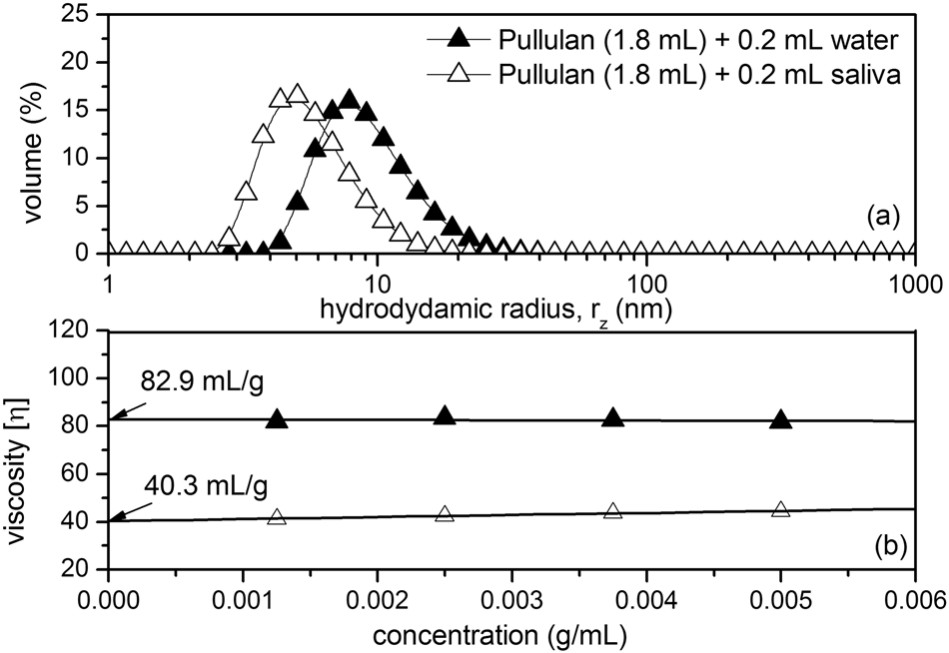
Changes in the apparent z-average hydrodynamic radius of pullulan before and after the addition of HSA **a** and Solomon–Ciuta results showing a change in the intrinsic viscosity of pullulan upon the addition of HSA **b**. DLS size distributions are given as an average of three measurements. Experiments performed at 20.0 °C, concentration of pullulan was 5 mgmL^−1^

**Fig. 3:**
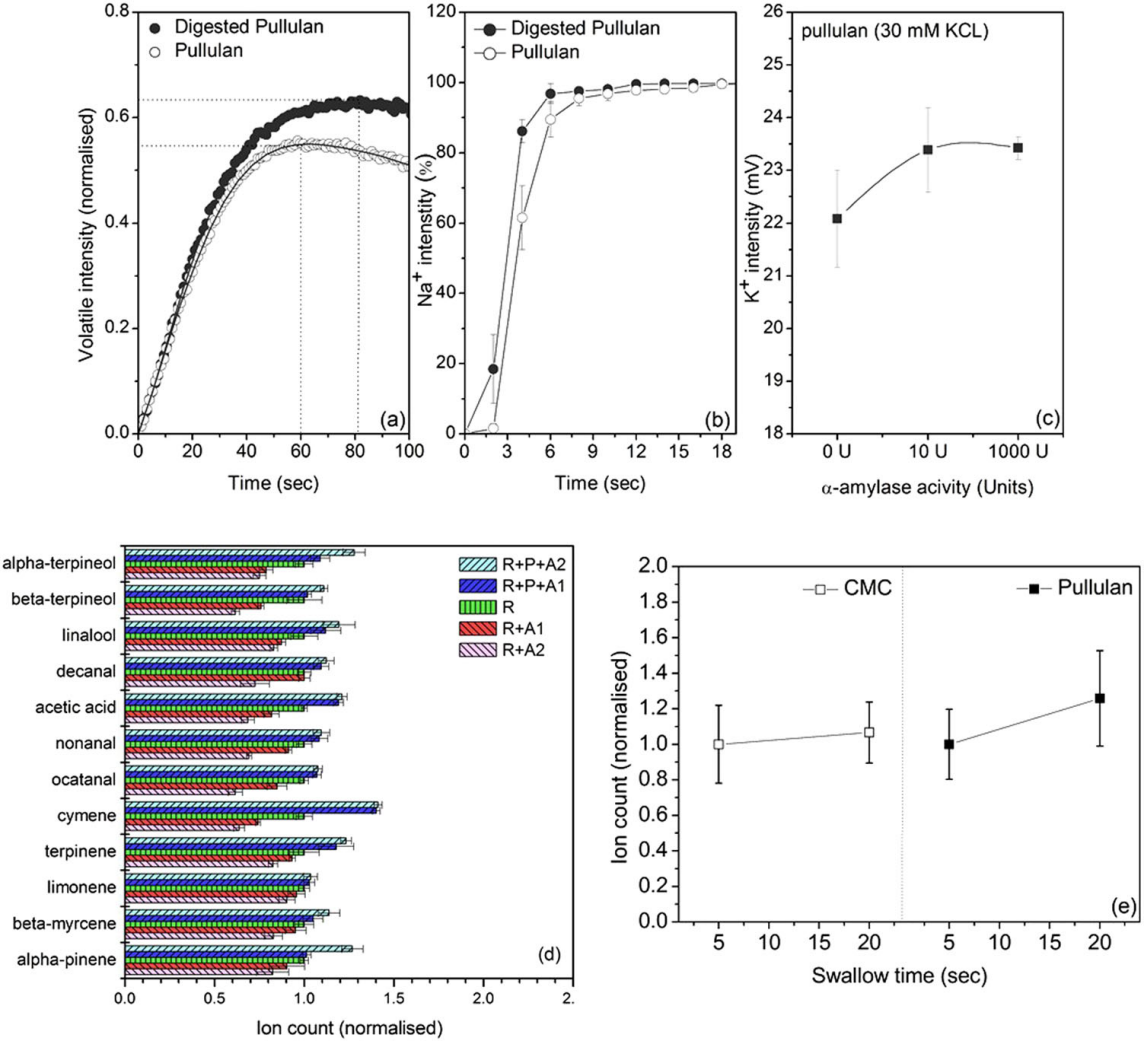
Results from APCI-MS, Na^+^ conductivity analysis and E-tongue showing the impact of polymer hydrolysis on the release of flavour from model solutions of pullulan, showing the real time data for α-ionone **a**, sodium ions **b** and potassium ions **c**, respectively; **d** GC–MS results showing the effect of pullulan hydrolysis on the relative headspace concentration of volatile aroma compounds from in Robinson’s orange squash, where ‘R’ represents the standard squash dilution, ‘A1’ and ‘A2’ are increasing α-amylase concentrations of 0.1 and 1 mgmL^−1^, and ‘P’ represents pullulan at a constant concentration of 2 mgmL^−1^; and **e** APCI in vivo analysis showing the comparative release of aroma compound ethyl butyrate from model drink solutions containing either pullulan or carboxy-methyl cellulose (CMC). Values are expressed as mean ± SD (*n* = 3)

Undigested pullulan showed an z- average apparent hydrodynamic radius, *r*_z_, of ~8.5 nm, which was not visible upon the addition of α-amylase, resulting in a z- average of ~5 nm (Fig. 2a). The addition of HSA also resulted in a two fold decrease in viscosity, as indicated by the Solomon-Ciuta extrapolation for intrinsic viscosity, [η] (Fig. 2b). Taken together, we can confirm that human salivary α-amylase is capable of partially digesting pullulan, producing smaller fragments of lower molecular weights and lower viscosity. The next important question became whether partial polymer hydrolysis correlates to an increase in the release of flavour from dilute systems. It is interesting to suggest that in thicker systems, such as starchy food, a decrease in the in mouth viscosity generated by the action of HSA is directly related to an enhanced flavour perception through a cross-modal interactions related to the perceived changes in mechanical stress.^20^ We therefore sought to analyse the release of taste and aroma compounds as a function of pullulan degradation. A selection of volatile aroma molecules used in this analysis were ethyl butyrate, hexanal, linalool, citral and α-ionone, while model taste compounds included sodium (Na^+^) and potassium (K^+^) ions. The results in Fig. 3a illustrate the release intensity and persistence of α-ionone from model solutions, before and after pullulan digestion. In the presence of undegraded pullulan solutions (4 mgmL^−1^), the headspace concentration for the majority of volatile aroma compounds reached a plateau, while it continued to increase for an additional ~20 s when the pullulan was digested by HSA. Although the time scale of this analysis is not representative of the very short amount of time needed to consume food and drink, the model system confirms the effect of pullulan digestion on aroma release. However, the rate of release may be increased during oral processing, unlike the current simulated in-vitro conditions. This is because, under real in mouth conditions, constant salivary secretion accompanied by mechanical changes due to mastication may enhance aroma release.^21^

A similar trend is observed in Fig. 3b, in which the conductivity analysis indicated an increase in the rate of release of sodium ions. This suggests that the availability of sodium can be increased by the oral degradation of pullulan. Similarly, in the next step we evaluated the intensity of potassium ions before and after enzyme hydrolysis, using the taste evaluation INSENT E-tongue (Fig. 3c). Although not statistically significant, results indicate that increasing the α-amylase concentration can increase the availability of K^+^ ions. The hypothesis was further tested in the presence of a commercial fruit drink. For its simplicity, we have chosen to analyse the effect of α-amylase hydrolysis on the release of aroma compounds from a dilute orange squash preparation ‘R’ in the presence and absence of pullulan ‘P’ (Fig. 3d). Interestingly, α-amylase (A), which is naturally present in saliva, reduced the headspace concentrations of the compounds, in a positive concentration dependent manner (A1, A2 in Fig. 3d). However, if pullulan is present, the aroma suppression of the orange squash is mitigated and the volatile aroma compounds are released into the headspace at higher concentrations. Furthermore, we performed in vivo simulations looking at the release of ethyl butyrate from a model drink containing sucrose and citric acid, in which we compared carboxymethylcellulose (CMC) with pullulan, corrected for viscosity (Fig. 3e). The retronasal ion intensity was recorded after swallowing the model drinks. For the drinks containing pullulan, it was observed that a late swallow (20 s) was correlated with a higher intensity of ethyl butyrate, compared to drinks containing CMC. Although results are not significantly different, this confirms our hypothesis that it is possible to maximise the release of flavour as a function of matrix viscosity, even in dilute solution conditions.

Given that α-amylase can be secreted to elevated concentrations during oral processing of food, our ex-vivo and in-vivo results are in excellent agreement and suggest that the release of aroma compounds can be enhanced in the presence of pullulan, despite the presence of other food constituents which might interfere with the normal functioning of the enzyme i.e. citric acids. However, as shown in Fig. 3, there are limitations in whether enzyme hydrolysis can significantly increase aroma release and perception in-vivo, in time for ingestion, which for some products, such as cordials or soft drinks, corresponds to only a couple of seconds. These simulations form the basis for our development of a mucoadhesive polymer system, which can be initiated by the action of the enzymes naturally present in the saliva. We suggest that by modifying the chemical properties of the polymer, such that it becomes adhesive towards the oral mucus, the loss of bioactive associated with the rapid ingestion can be mitigated.

### Developing a functional mucoadhesive pullulan analogue

**Fig. 4:**
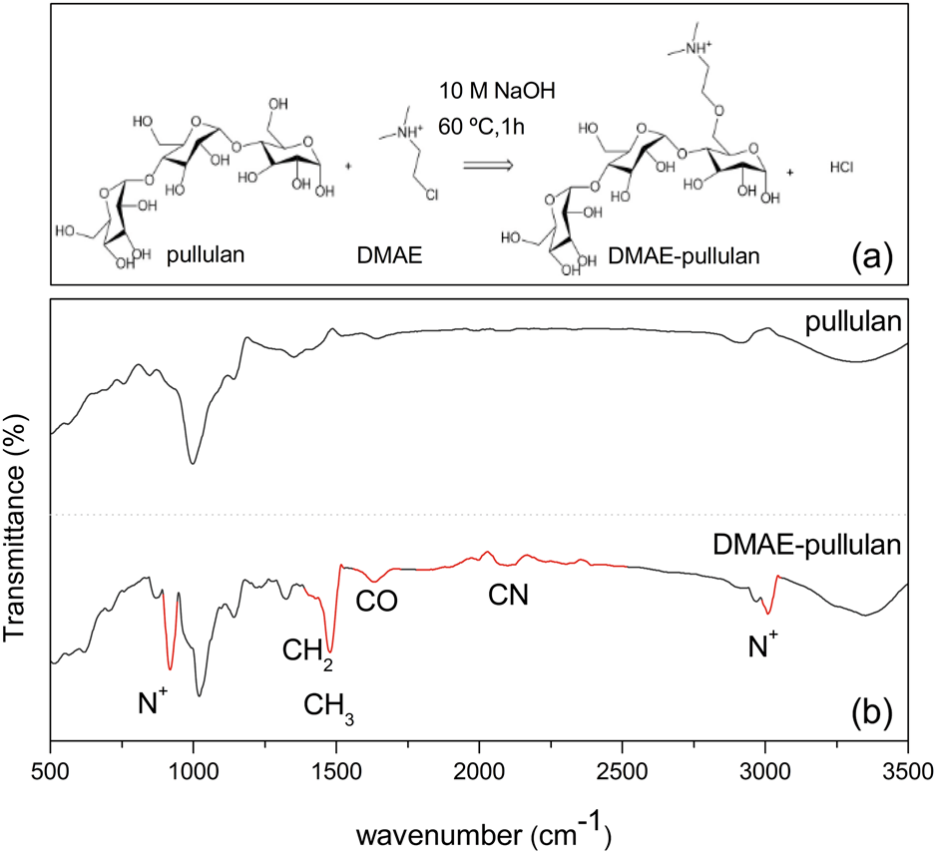
Schematic representation of the chemical modification of pullulan showing the addition of the tertiary amine, dimethylaminoethyl (DMAE) chloride **a** and FT-IR spectra of pullulan before after synthesis highlighting the qualitative changes in the spectral intensity correlating to the new functional groups **b**. The reaction was performed using an adapted version from San Juan et al.^31^ Five gram of Pullulan (Carbosynth, 200 kDa) was dissolved in 25 ml of distilled water and mixed with a 25 mL 10 M sodium hydroxide solution to activate the pullulan hydroxyl functions. Then, 35gm of 2-chloro-N,N dimethylethylamine hydrochloride was added to the mixture and left stirring at 60 °C for 1 h. After the reaction was completed, the mixture was washed four times with 50 ml diethyl ether and after was diluted in water to a concentration of 10 mgmL^−1^ and adjusted to pH 7 using HCl

Initially our studies began with the coupling of amino functional groups onto the polysaccharide backbone to produce a functional cationic pullulan analogue, without impeding access for enzyme hydrolysis. The most promising candidate was for dimethylaminoethyl-pullulan (DMAE-pullulan), synthesised, as shown in Fig. 4a. The polymer was purified and the resulting material analysed by FT-IR (Fig. 4b). In comparison with the unmodified pullulan spectra, the absorption bands detected at ~900 and ~3050 cm^−1^ correspond to stretching and wagging vibrations of the amino group while the strong absorption at 1390 and 1460 cm^−1^ correspond to the CH_2_ and CH_3_ vibrations of the dimethylaminoethyl chain. A characteristic CO group is observed around 1720 cm^−1^, while broader and weaker vibrations are observed in the region 1800–2500 cm^−1^, which indicate the presence of the CN bonds of the amino group (Fig. 4b). Therefore, the results indicate that the DMAE group was grafted onto the pullulan backbone.

**Fig. 5:**
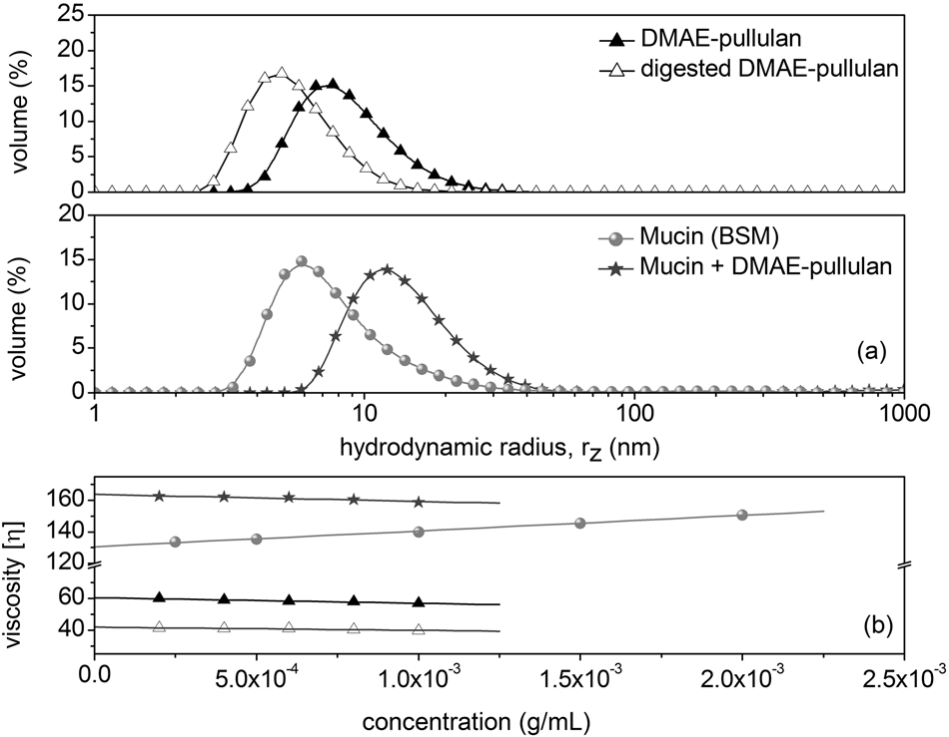
Results showing changes in the apparent z-average hydrodynamic radii of DMAE-pullulan, mucin, α-amylase, and the result of their interactions **a**, and viscosity results showing the Solomon-Ciuta estimations of the intrinsic viscosities of DMAE-pullulan, mucin, α-amylase, and their mixtures **b**. The concentrations represent dilutions of each sample. DLS size distributions are given as an average of three measurements. Performed at 20.0 °C, macromolecular concentrations were in a ratio of 1:1

Then, our next goal was to evaluate the ability of the newly modified cationic pullulan to interact with our two main salivary components, mucin and α-amylase. First, a combined viscosity and particle size analysis approach has confirmed the ability of α-amylase to reduce the hydrodynamic particle size (radius) of the newly modified polymer from ~8 to ~5 nm (Fig. 5a-top). In the presence of submaxillary mucin (~6.5 nm) an increase in a particle size distribution was observed, suggesting mucoadhesive phenomena, corresponding to a z-average hydrodynamic radius of r_z_ of ~12 nm (Fig. 5a-bottom). Similarly, we evaluated changes in the intrinsic viscosity (Fig. 5b), which corresponded to a 32% decrease upon the addition of α-amylase. By contrast, the intrinsic viscosity of the DMAE-mucin mixture was 23% higher than the viscosity of submaxillary mucin (Fig. 5b).

**Fig. 6:**
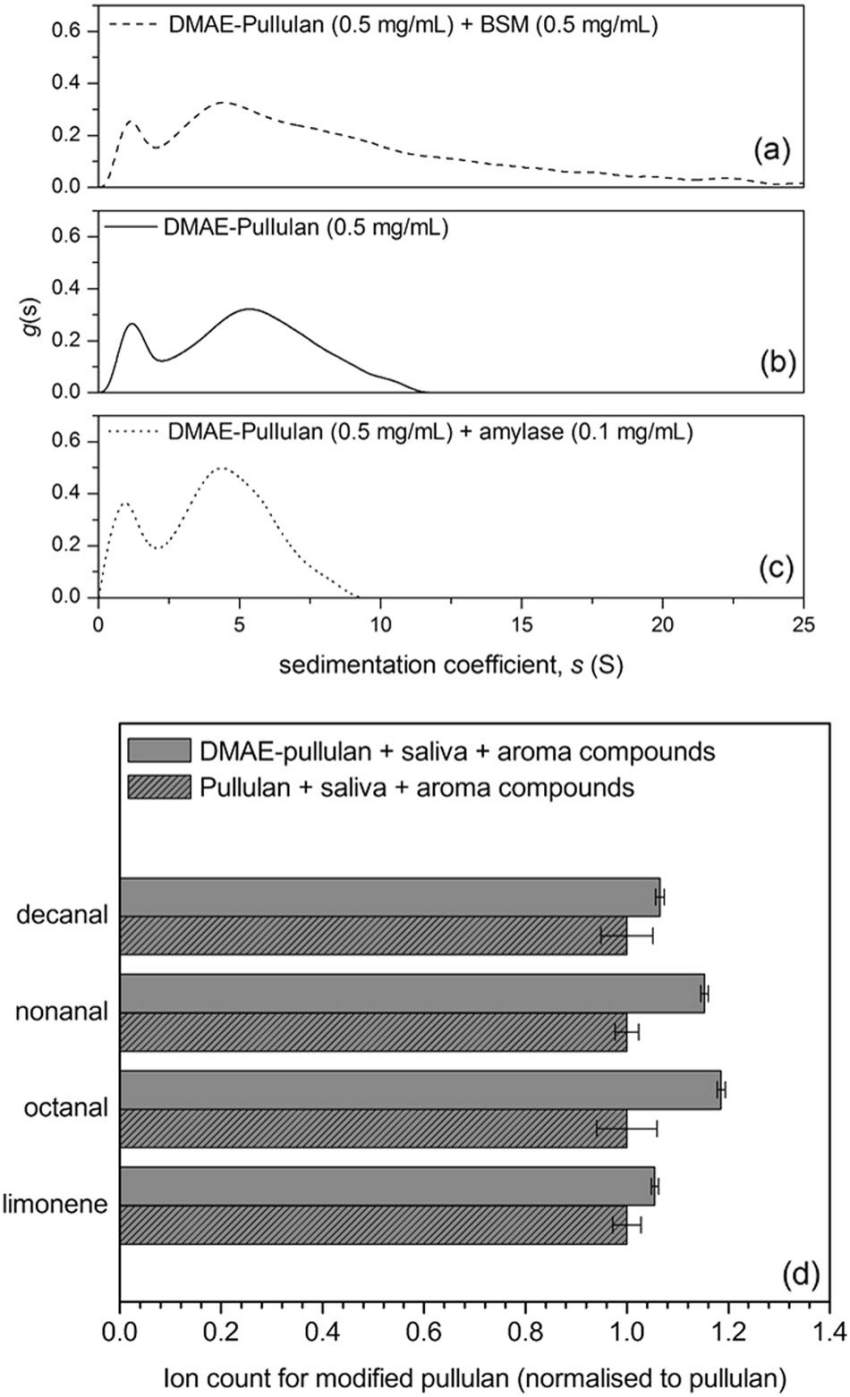
Sedimentation velocity, *g*(s) analysis showing the sedimentation coefficient distributions for DMAE-pullulan at 0.5 mgmL^−1^ **b** and the result of the addition of mucin at 0.5 mgmL^−1^ **a** and α-amylase at 0.1 mgmL^−1^ **c**; and the GC–MS volatile analysis from modified and unmodified pullulan and solutions upon the addition of saliva **d**. Rotor speed: 45,000 rpm (130,000 × *g*), 20.0 °C. The distributions reflect the real time migration of molecules driven by the centrifugal force. For the same type of macromolecule, i.e. DMAE-pullulan, a larger S value corresponds to a larger molecular weight

The interaction analysis was further reinforced by a SV–AUC interactions experiment which allowed us to directly monitor changes in the sedimentation coefficient distribution of DMAE-pullulan upon the addition of α-amylase and submaxillary mucin (Fig. 6). By itself, DMAE-pullulan revealed a rather broad macromolecular sedimentation profile, indicative of a heterogeneous composition, but nearly identical to the native unfractionated food grade pullulan used for the synthesis, which confirms that the chemical synthesis did not cause the polymer to degrade. At a constant concentration of 0.5 mgmL^−1^, the sedimentation coefficient distribution ranged from 1S to ~12S (Fig. 6b). An initial assessment reveals an increase in the sedimentation coefficient distribution to ~25S upon the addition of mucin, indicative of an interaction, although a large proportion of sedimentation species (~70%) remained the same (Fig. 6a). One of the methods previously used to assess for mucoadhesion is measuring the sedimentation coefficient distribution ratio of the mucin/polymer complex to that of the mucin (*s*_complex_/*s*_mucin_)^2^. Our results showed that the ratio ranged from 1.1 to 2 (Fig. 6a). These values are similar to DEAE-dextran which are still fairly modest compared to stronger mucoadhesive polymers such as chitosan, which has been shown to give sedimentation ratios of up to ~40. However, chitosan mucoadhesion is an extreme example which would not only lead to the precipitation of mucin glycoproteins, but also other anionic glycoproteins present in the saliva, causing a very unpleasant astringent sensation. Overall, our results demonstrate that up to 30% of DMAE-pullulan can bind mucin, as given by the area under the sedimentation curve (Fig. 6a).

By contrast, the addition of α-amylase caused a reduction in the sedimentation coefficient distribution of the modified polymer from ~12S to ~9S (Fig. 6c). This translates to a ~25% loss in higher z-average molecular weight fractions, and an increase in the concentration of lower molecular weight DMAE-pullulan fractions. These values are qualitatively consistent with the results from viscosity and DLS (Fig. 5). In addition, we have compared the aroma release ability of the modified polymer to its native pullulan counterpart in a model ex-vivo system containing saliva and aroma compounds (Fig. 6d). To our surprise, it was shown that the release of the volatiles was significantly increased in the presence of DMAE-pullulan, as opposed to pullulan. The additional increase can be explained by the loss in molecular weight and viscosity of the modified polymer, and perhaps due to a reduction in the damping effects other proteins present in the saliva.

It is worth mentioning that our preliminary results tentatively indicate that the interaction mechanisms of DMAE-pullulan with saliva may lead to very minor changes in the in mouth rheology of the bolus, since the viscosity increase due to adhesive interactions is counterbalanced by the degree of hydrolysis. As a result, the sensory properties of the food/saliva mixture, i.e. mouthfeel, are expected to be the same. However, we would further need to perform in-vivo trials and take into account factors such as mastication and salivation, which have been shown to play a key role in the release of volatile aroma compounds.^21^ Although this would be more applicable to solid food systems where the breakdown of the food structure which can influence the rate of release of aroma compounds, as well as altering the proportions of hydrophilic compounds.^21^ Similarly, mucoadhesion may play a role in the after taste, by increasing the residence time of flavour compounds onto the oral surface. Though in order to analyse this effect we would first require an approved food grade cationic pullulan analogue.

Research is currently being undertaken to identify greener ways to produce cationic pullulan analogues that would meet the required quality and purity criteria of food ingredients, but we suggest that a food grade cationic pullulan could become one of the next generation mucoadhesive biopolymer candidates targeted at the oral cavity. Regardless of the final chemical product and instrumental analysis, we must remember that flavour is not just a group of attributes or a group of chemicals, but a perceptual phenomenon that will strongly depend on the physiological status of the individual.

## Discussion

The oral processing simulation experiments have shown that pullulan can be used for the targeted release of bioactive flavour compounds, as a result its partial in-vivo digestion. The time scale of polymer hydrolysis was over 20 s, however for a lot of liquid and semi-liquid foods such as juices or yoghurts, the oral transit time is no longer than a few seconds which results in a rapid loss of flavour through ingestion. To address this issue, we have synthesised a cationic pullulan analogue, DMAE-pullulan, which was assessed for its mucoadhesive ability, whilst ensuring it retains its inherent susceptibility to α-amylase hydrolysis. We have shown that the cationic polymer binds to submaxillary mucin, aimed at increasing the oral retention of flavour compounds. Then, we have shown that the release of flavour compounds can be enhanced through the action of salivary α-amylase, which partially degrades the modified polymer. Once a food grade cationic pullulan becomes available, sensory experiments would add to our analysis and provide a broader explanation of its impact on flavour perception.

To conclude, we developed a unique concept of a controlled release mucoadhesive system targeted for the oral cavity which may have strong resonances for enhancing the release of flavour and other bioactive compounds during oral processing.

## Methods

### Sample preparation

Bovine submaxillary mucin (type I-S, M3895), human salivary α-amylase (type IX-A, A0521), 200 kDa pullulan standard (01615) and volatile aroma compounds used in this study were purchased from Sigma Aldrich (Dorset, UK). The food grade 200 kDa sample was purchased from Carbosynth, UK. The 0.1 M phosphate buffered saline (PBS) was made according to Green (1933),^22^ (Fisher Scientific, UK). Saliva samples were from the Centre for Biomolecular Sciences, University of Nottingham. All samples were collected in accordance with the ethical approval R12122013, Faculty of Medicine and Health Sciences Research Ethics Committee, Queens Medical Centre, Nottingham University Hospitals.^23^ Participation was voluntary and informed written consent was obtained. All data were held in accordance with the Data Protection Act. The pooled samples were centrifuged (6000 g), dialysed against 0.1 M phosphate chloride bugger using a 14 kDa dialysis membrane and filtered through a 0.45 µm membrane filter to remove larger aggregates, such as gelled mucus and small molecular weight peptides, respectively, then stored at −80 °C until use. Loading and unloading of samples was carried out in a Level 2 microbiological safety cabinet.

### Orange squash

Robinson’s sugar free orange squash concentrate was purchased from the local supermarket. Final samples used for the GC analysis were diluted according to the manufacturer, one part concentrate and four parts water/ solution. The samples were mixed with the polymer solutions such that the concentration of squash is always constant. Highly purified RO (reverse osmosis) water was used throughout the sample preparation.

### Sedimentation Velocity-Analytical ultracentrifugation (SV-AUC)

Experiments were performed at 20.0 °C using the Optima XL-I analytical ultracentrifuge (Beckman, Palo Alto, USA) equipped with Rayleigh interference optics. Samples of 395 μL (and 405 μL solvent) were injected into the 12 mm double sector epoxy cells with sapphire windows and run at 40,000 rpm (120,000 × *g*). Scans were taken at 2 min intervals. The interference system produced data derived by recording changes in concentration (in fringe units) versus radial displacement. The results were analysed in SEDFIT using the least squares ls-g*(s) or ‘*g*(s)’ and the diffusion corrected c(s) processing methods (the latter valid because of the high degree of fractionation/low polydispersity of the P200 pullulan), by generating sedimentation coefficient distributions, s_20,w_ (in Svedberg units, S = 10–13 s) normalised to standard conditions (viscosity & density of 0.1 M PBS at 20.0 °C).^24–26^

### Gas chromatography–mass spectrometry (GC–MS)

The Trace 1300 series Gas Chromatograph coupled with the single-quadrupole mass spectrometer (Thermo Fisher Scientific, Hemel Hempstead, UK) was used. Samples were incubated at 37.0 °C for 20 min with intermittent stirring. Then, the solid phase microextraction (SPME) fibre (50/30 μm DVB/CAR/PDMS, Supelco, Sigma Aldrich, UK) was used to extract for 40 min then desorb for 1 min. Separation was carried out by a ZB-WAX capillary gas chromatography column (length 30 m, internal diameter 1 mm, 1.00 μm film thickness). The column temperature was initially at 40.0 °C for 2 min, then increased by 6.0 °C every minute up until 250.0 °C and held for 5 min. Full scan mode was chosen to measure volatile compounds (mass range from 20 to 300 Da). A splitless mode was used, and a constant carrier pressure of 18 psi was applied. Volatiles were identified by comparison of each mass spectrum with either the spectra from the *NIST* Mass Spectral Library.

### Atmospheric Pressure Chemical Ionization-Mass Spectrometry (APCI-MS)

The APCI-MS (Platform II, Micromass, Manchester) was used to analyse the real time concentration of volatile compounds under static conditions. A final concentration of ~10–50 ppm (parts per million) was sampled with an air flow adjusted to 50 ml/min. The instrument was set in Selective Ion Recording (SIR) mode to monitor the selected mass to charge ions (m/z). The ion intensity was measured at cone voltage of 50 V, source temperature of 75 °C and dwell time of 0.02 s. The in-vivo analysis shown in Fig. 3d was performed by consuming model drink solutions of sucrose, citric acid in which pullulan or CMC were added, and the retronasal ion intensity was captured by exhaling into the MS-NOSE interface of the APCI.

Sampling took place until the signal plateaued and started to decrease. The curves were integrated in Mass Lynx^TM^ (Waters, UK).

### Dynamic Light Scattering (DLS)

The experiments were performed using the Zetasizer Nano-ZS detector and low volume (ZEN0112) disposable sizing cuvettes (Malvern Instruments Ltd, Malvern, UK). The samples were measured at (20.00 ± 0.01)°C using the 173° scattering angle collected for 3 runs of 10 s. For polydisperse particles, DLS can provide useful information about the size (radius) of molecules by calculating an estimate for z-average hydrodynamic radius, *r*_z_, and translational diffusion coefficient, D_*trans*_, via the Stokes–Einstein equation:

1 $${\mathrm{D}}_{{\mathrm{trans}}} = \frac{{k_BT}}{{3\pi \eta d}}$$

where the hydrodynamic diameter *d* = 2r_z_, k_*B*_ is the Boltzmann constant, η is the solvent viscosity, T is absolute temperature (K), and D_trans_ (cm^2^s^−1^). The contribution of rotational diffusion effects to the autocorrelation function is assumed negligible (see Burchard, 1992).^7^

### Capillary viscometry

Flow times of solvent (*t*_0_) and solutions (*t*_s_) were measured using a semi-automated (Schott Geräte, Hofheim, Germany) U-tube Ostwald capillary viscometer immersed in a temperature controlled water bath at 20.0 °C. A constant volume of 2.0 ml was sampled at a series of mucin concentrations (0.2–1.0 mgmL^−1^), sufficiently low to allow the assumption that no correction was needed for solution density, assuming η_s_/η_0_ is equal to *t*_s_/*t*_0_. The intrinsic viscosity [η] plot is shown as according to the Solomon–Ciuta equation and extrapolated to zero concentration to account for non-ideality.^27,28^

### E-tongue

Digested and undigested pullulan solutions were made in 30 mM potassium chloride buffer and poured into the sample cups for the electronic tongue in triplicate (Taste Sensing System TS-5000Z). Manufactures guidelines were used for analysis and data extraction. The experimental design was kindly performed by New Food Innovation specialists as in previous studies.^29,30^

### Conductivity metre

Dissolution of sodium was evaluated using a Mettler Toledo conductivity metre (Ohio, USA). A 1 ml sodium chloride solution (0.1 mgmL^−1^) was dissolved in a beaker containing normal and digested pullulan solution. Data were recorded every 2 s until a plateau was reached (~20 s). For this analysis, a 1 mL solution of sodium was added at a concentration of 1 mgmL^−1^ into a 50 ml pullulan solution, equivalent to the dissolution of 0.2 mg of sodium, under constant magnetic stirring and maintained at 25.0 °C. Three replicates were performed and normalised by conductivity.

### Synthesis of dimethylaminoethyl (DMAE) pullulan

The reaction was performed using an adapted version from San Juan et al.^31^ Five gram of Pullulan (Carbosynth, 200 kDa) was dissolved in 25 ml of distilled water and mixed with a 25 mL 10 M sodium hydroxide solution to activate the pullulan hydroxyl functions. Then, 35gm of 2-chloro-N,N dimethylethylamine hydrochloride was added to the mixture and left stirring at 60 °C for 1 h. After the reaction was completed, the mixture was washed four times with 50 mL diethyl ether and after was diluted in water to a concentration of 10 mgmL^−1^ and adjusted to pH 7 using HCl. The solution was further cleaned of organic solvents and concentrated in a rotary evaporator, after which was dialysed in PBS buffer on a 14,000 Da (g/mol) membrane for two days. The resulting solution was freeze-dried which resulted in the formation of white odourless powder. The powder was stored at 4 °C until needed.

### Fourier-transform infrared spectroscopy (FT-IR)

The resulting powder was subjected to FT-IR analysis. Measurements were performed in transmission mode on an IRAFFINITY-1S spectrometer equipped with an A219653 attenuated total reflection (ATR) module (Shimadzu, Japan). For each sample, the spectrum was taken as the average of three different measurements at various sites of the dry sample Spectra were acquired between 500 and 3500 cm^−1^ at a resolution of 4 cm^−1^. Dry pullulan samples were pressed against the diamond surface to ensure good contact. Measurements were repeated twice for reliability.

### Statistical analysis

GC–MS and conductivity samples were analysed in triplicate in a randomised sample order, and the error is given as a as mean ± SD (*n* = 3). Figures were made in Origin 7.5 (OriginLab, USA).

### Reporting Summary

Further information on research design is available in the Nature Research Reporting Summary linked to this article.

## Supplementary information

Reporting Summary

## Data Availability

The data that support the findings of this study are available from the corresponding author upon request.

## Supplementary information

**Supplementary information** accompanies the paper on the *npj Science of Food* website (10.1038/s41538-019-0043-y).
